# The efficacy and cost‐effectiveness of a family‐based economic empowerment intervention (Suubi + Adherence) on suppression of HIV viral loads among adolescents living with HIV: results from a Cluster Randomized Controlled Trial in southern Uganda

**DOI:** 10.1002/jia2.25752

**Published:** 2021-06-27

**Authors:** Yesim Tozan, Ariadna Capasso, Sicong Sun, Torsten B Neilands, Christopher Damulira, Flavia Namuwonge, Gertrude Nakigozi, Abel Mwebembezi, Barbara Mukasa, Ozge Sensoy Bahar, Proscovia Nabunya, Claude A Mellins, Mary M McKay, Fred M Ssewamala

**Affiliations:** ^1^ School of Global Public Health New York University New York NY USA; ^2^ Brown School Washington University in Saint Louis Saint Louis MO USA; ^3^ International Center for Child Health and Development Brown School Washington University in St. Louis St. Louis MO USA; ^4^ Center for AIDS Prevention Studies School of Medicine University of California San Francisco CA USA; ^5^ International Center for Child Health and Development Washington University in St. Louis Uganda Field Office Masaka Uganda; ^6^ Rakai Health Sciences Program Kalisizo Uganda; ^7^ Reach the Youth (RTY) Uganda Kampala Uganda; ^8^ Mildmay Uganda Kampala Uganda; ^9^ HIV Center for Clinical and Behavioral Studies Department of Psychiatry New York State Psychiatric Institute and Columbia University New York NY USA

**Keywords:** HIV, ART, SUUBI, Cost‐effectiveness analysis, Uganda, adolescents, economic empowerment, savings‐led intervention

## Abstract

**Introduction:**

Evidence from low‐resource settings indicates that economic insecurity is a major barrier to HIV treatment adherence. Economic empowerment (EE) interventions have the potential to improve adherence outcomes among adolescents living with HIV (ALWHIV) by mitigating the effects of poverty. This study aims to assess the efficacy and cost‐effectiveness of a savings‐led family‐based EE intervention, Suubi + Adherence, aimed at improving antiretroviral therapy (ART) adherence outcomes ALWHIV in Uganda.

**Methods:**

Adolescents (mean age 12 years at enrolment; 56% female) receiving ART for HIV at 39 health centres were randomized to Suubi + Adherence intervention (n = 358) or bolstered standard of care (BSOC; n = 344). A difference‐in‐differences analysis was employed to assess the change in the proportion of virally suppressed adolescents (HIV RNA viral load <40 copies/mL) over 24 months. The cost‐effectiveness analysis examined how much the intervention cost to virally suppress one additional adolescent relative to BSOC from the healthcare provider perspective.

**Results:**

At 24 months, the intervention was associated with an 8.85‐percentage point [95% confidence interval (CI) 0.80 to 16.90 percentage points] increase in the proportion of virally suppressed adolescents between the study arms (*p* = 0.032). Per‐participant costs were US$177 and US$263 for the BSOC and intervention groups respectively. The incremental cost of virally suppressing one additional adolescent was estimated at US$970 [95% CI, US$508 to 10,725] over two years.

**Conclusions:**

Our results support the integration of family‐based EE interventions into adherence‐support strategies as part of routine HIV care in low‐resource settings to address the underlying economic drivers of poor ART adherence among ALWHIV. Moreover, per‐participant costs to achieve viral suppression do not seem prohibitive compared to other community‐based adherence interventions targeted at ALWHIV in low‐resource settings. Further research on combination interventions at the nexus of economic security and HIV treatment and care is needed to inform the development of feasible and scalable HIV policies and programmes.

## 1 Introduction

Despite drastic improvements in the health of people living with HIV and AIDS (PLWHA) with increased access to antiretroviral therapy (ART), HIV/AIDS remains a serious public health problem in sub‐Saharan Africa (SSA) [1], particularly in children aged under 19 years in the region [2]. While increasing numbers of perinatally HIV‐infected children survive into adolescence with treatment, adolescents living with HIV (ALWHIV) are the only age group with increased mortality rates due to HIV/AIDS in SSA [3]. This trend can, in part, be explained by the fact that adolescents are less adherent to treatment for chronic health conditions [4], including HIV [3, 5], and have higher attrition rates from HIV treatment and care, with higher rates of treatment failure compared to younger children and adults [5]. While the most obvious implication of suboptimal ART adherence in HIV patients is an immunological decline (lower CD4 cell counts) and disease progression because of unsuppressed viral load (VL), adherence is also critical to maximizing the preventive effect of HIV treatment and slowing the spread of ART resistance at the population level [6]. Adolescents and young people are, hence, the primary target population in HIV programmes for achieving the ambitious 95‐95‐95 targets established by the Joint United Nations Programme on HIV/AIDS by 2030 [7].

Studies have shown that VL is a better predictor of HIV morbidity and mortality among PLWHA compared to CD4 cell count [8, 9] and a risk factor for opportunistic infections, irrespective of CD4 cell count [9]. An undetectable VL, defined as having less than 40 copies of the virus in one millilitre of blood, has clinical significance with the risk of viral rebound being higher among PLWHA with detectable VL [10]. Lastly, evidence shows that undetectable VL drastically reduces the risk of onward HIV transmission [6]. Given the clinical relevance of VL, the global target is to virally suppress 95% of all people receiving ART by 2030 [7].

Suboptimal adherence to treatment for chronic health conditions has proven to be an intractable public health problem [11]. Among ALWHIV, multi‐level factors, including mental health, substance use, family/social support, economic insecurity and ART regimen complexity, play salient roles in ART adherence [12]. For ALWHIV, the World Health Organization (WHO) recommends community‐based interventions involving home visits, educational activities, counselling, mentoring and peer leadership, to promote their overall wellbeing, adherence and retention in care [13, 14, 15]. In poverty‐stricken communities, economic insecurity that results in lack of money for transportation to clinics for routine follow‐ups, inability to secure adequate nutrition to adhere to HIV care recommendations and prioritization of financial resources towards food, education and other basic needs, are shown to be a risk factor for suboptimal ART adherence among ALWHIV [13, 16]. Evidence from low‐resource settings indicates that combination interventions that integrate traditional health education with economic empowerment (EE) interventions, including incentivized financial savings directed towards specific purposes such as education and family income‐generating activities (IGAs), can significantly improve the health and developmental outcomes of vulnerable adolescents [16, 17, 18]. However, there is limited evidence on their efficacy and cost‐effectiveness [18].

Uganda is among the countries highly affected by HIV/AIDS in SSA. The 2018 Uganda national statistics indicate an HIV prevalence of 5.7% among persons aged 15 to 49 years [19]. There are approximately 170,000 youth living with HIV in the country, and HIV is expected to continue to affect the youth disproportionately because of this subgroup’s high socioeconomic vulnerability [20] and poor ART adherence [3, 21]. In this context, there is a pressing need for innovative interventions that promote behaviour change among ALWHIV, while simultaneously strengthening families’ ability to sustain these changes by enhancing their economic capabilities. This study presents the efficacy and cost‐effectiveness of a savings‐led family‐based EE intervention, titled Suubi + Adherence, aimed at improving ART adherence among ALWHIV in southern Uganda [22].

## 2 Methods

### 2.1 Trial population and setting

The Suubi + Adherence study was a two‐arm cluster randomized controlled trial (RCT; NCT#01790373) to assess the efficacy of a savings‐led family‐based EE intervention on ART adherence among ALWHIV in five districts in Uganda. The trial is described in detail elsewhere [22]. Briefly, adolescents (mean age 12 years at enrolment; 56% female) receiving HIV treatment at 39 health centres were randomized to Suubi + Adherence intervention (n = 358) or bolstered standard of care (BSOC; n = 344). From September 2013 through January 2018, trained field staff collected biomarker laboratory data (VL) and administrative data (pill counts and pharmacy refills) and conducted in‐person interviews with participating adolescents. The inclusion criteria for participants were as follows: (1) being 10 to 16 years of age at enrolment; (2) being HIV‐positive and aware of status; (3) having been prescribed ART; (4) being registered at one of the participating health centres approved by the Ugandan Ministry of Health (MOH) to dispense/administer ART and (5) living with families, not institutions – as the two groups may have different needs regarding ART adherence.

Participants in both study arms received BSOC at participating health centres, involving medical care and psychosocial support on ART resistance and adherence from trained lay counsellors and expert clients (i.e. peer navigators) along with Uganda‐MOH produced communication materials for families around these topics. Participants in the intervention arm also received six additional counselling sessions on HIV and ART adherence and mentorship from peers/research assistants; four workshops on asset building, IGAs and financial saving and planning from the non‐governmental partner, Reach the Youth‐Uganda; and an incentivized savings account (child development account [CDA]) to which the study provided an initial deposit and matched the adolescent’s monthly savings at a ratio of 1:1 for 24 months. Parents and relatives of participants were encouraged to deposit money into CDAs, which were housed at local financial institutions. Matched funds could only be used for education‐related expenses, IGAs, or verifiable healthcare expenses. All activities were held at participating health centres and primarily facilitated by lay counsellors and programme staff. The intervention was implemented over 24 months.

All caregivers provided written consent, and all adolescents assented to study participation. All study procedures were approved by the institutional review boards of Columbia University (IRB‐AAAK3852), Makerere University School of Public Health (IRB‐210), and Uganda National Council for Science and Technology (SS2969).

### 2.2 Outcomes

The primary study outcome was the proportion of virally suppressed adolescents at 24‐months. Viral suppression was defined as an HIV RNA VL of <40 copies/mL. The Rakai Health Sciences Program conducted VL testing at each of the two data collection time points. Plasma VL measurements were quantified in blood samples collected in EDTA tubes using Abbott Real Time HIV‐1 RNA PCR, version 5.00. In accordance with the Abbott Real Time assay’s sensitivity, where 40 copies/mL was the lowest detectable value and hence the most uniform indicator of viral suppression, VL was dichotomized as undetectable (<40 copies/mL) or detectable (≥40 copies/mL) [10].

### 2.3 Statistical analysis

Data collected at baseline and 24‐month follow‐up were used to assess intervention efficacy using an intent‐to‐treat (ITT) approach. Rao–Scott chi‐square tests were first used to assess if the changes over time in the proportion of virally suppressed adolescents were statistically significant in both study arms. We conducted a difference‐in‐differences analysis [23, 24] to compare the changes in the proportion of virally suppressed adolescents by study arm at 24 months. Robust standard errors were clustered at the health centre level to account for the intra‐group correlation within each centre. The key assumption of the difference‐in‐differences estimation was that, in the absence of intervention, BSOC and intervention groups would have experienced the same average change in the proportion of virally suppressed adolescents over time. Because we used data from a clustered RCT and there were no significant differences in sociodemographic and HIV‐related characteristics by study arm at baseline (Table S1), the assumption of the parallel trend was not violated. Listwise deletion was employed for missing data on the primary outcome measure. We also conducted Treatment‐on‐the Treated (TOT) analysis only on intervention participants as per CONSORT diagram. All statistical analyses were performed in Stata 15 (StataCorp LP, College Station, TX, USA).

### 2.4 Cost analysis

Per‐participant costs were estimated for each study arm using an activity‐based micro‐costing approach (See Table S2 for a breakdown of costs). We measured and valued all resources used for each activity in the BSOC and intervention arms. Activities included recruitment of participants, health education sessions and mentorship, IGA/microenterprise workshops, contribution to matched CDAs, stakeholder engagement and dissemination and programme monitoring and evaluation. Costs were carefully recorded throughout the trial and extracted from the programme’s administrative records. Time costs of programme staff were estimated and apportioned according to time devoted to programme activities in each study arm. Other recurring implementation costs over the study period included training of programme staff and implementing partners (lay counsellors and expert clients/peer navigators), facilitation/transportation refund (lay counsellors, expert clients/peer navigators and health centre directors) for programme‐related activities, transportation refund (community extension workers responsible for small business and financial literacy training), communication (airtime for phones used for community mobilization by lay counsellors and expert clients/peer navigators), transportation by programme staff (fuel to health centres, car hire), transport refund for participants attending intervention‐related activities at health centres, office supplies and printing and other miscellaneous expenses. Space donated at participating health centres and time donated by lay counsellors and bank personnel for programme‐related activities were measured and valued to arrive at the economic costs of the intervention. Research costs, including the purchase of Wisepill devices (used for capturing adherence data in real‐time), home visits for pill counts and follow‐up assessments and VL testing, were excluded from the cost analysis. The treatment‐of‐the‐treated (TOT) sample was used to calculate per‐participant costs conservatively for each cost category. For comparison, we present per‐participant costs estimated using the ITT sample in Supplementary Material. All costs were summed to estimate the total per‐participant cost for each study arm over the 24‐month intervention period. Costs were adjusted for inflation using the Ugandan Consumer Price Index in Masaka [25] and discounted at an annual rate of 3% to the start year of the trial, and expressed in 2015 US dollars [26].

### 2.5 Cost‐effectiveness analysis

This cost‐effectiveness analysis used the costing data collected during the 24‐month intervention period and the primary outcome data from baseline and 24‐month follow‐up. The analysis examined how much the intervention cost to virally suppress one additional adolescent relative to the BSOC from the healthcare provider perspective and centred on the computation of incremental cost‐effectiveness ratios (ICERs). First, we calculated the cost difference between the intervention and BSOC arms based on total per‐participant costs for each arm. Second, we calculated the number of additional virally suppressed adolescents over the intervention period based on the estimated net change in the proportion of adolescents with an undetectable VL across each study arm. Third, we estimated the ICERs by dividing the incremental total cost of providing the intervention to participating adolescents in the intervention arm by the number of additional virally suppressed adolescents to compute the incremental cost of virally suppressing one additional adolescent. We also conducted a sensitivity analysis [27] in which we computed the ICERs for a pessimistic scenario (high cost/low effectiveness) and an optimistic scenario (low cost/high effectiveness) for the intervention. The range for intervention effectiveness was based on the 95% confidence intervals of the primary outcome measure, and we varied the estimated per‐participant cost difference by 20%. Reporting of the cost‐effectiveness analysis followed the Consolidated Health Economic Evaluation Reporting Standards (CHEERS) [28].

## 3 Results

### 3.1 Participant demographics

Participant recruitment began in July 2012. Data collection and intervention implementation commenced in January 2014 and the study ended in December 2015. Table S1 presents participants’ baseline demographic characteristics; 56.41% of participants were female, the average age was 12.42 years, 25.93% were double orphans and 38.60% were single orphans. The average time since participants knowing their HIV status was 4.18 years. About 47% reported having a parent as primary caregiver, and most (87.32%) were enrolled in school at baseline. The mean household size was 6. There were 414 participants (58.97%) who had undetectable VL at baseline. Assessments were conducted at baseline and every 12 months thereafter, up to 4 years [29].

Over 24 months, attrition due to loss to follow‐up, withdrawal from the study, or death was relatively consistent across study arms; attrition was 4.7% and 6.5% for BSOC and intervention arms respectively (Figure S1). At 24‐month follow‐up, both BSOC (n = 6; 1.7%) and intervention groups (n = 4; 1.1%) had VL values missing for reasons other than attrition. However, Rao–Scott chi‐square tests indicated no significant differences in attrition rates and missing VL values between study arms (*F* = 0.34, *df* = 1, *p* = 0.56).

### 3.2 Intervention efficacy

Table 1 presents the difference‐in‐differences analysis results examining viral suppression by the study arm. A total of 203 adolescents in the BSOC group and 218 in the intervention group were virally suppressed at the end of the trial. The change in the proportion of virally suppressed adolescents in the BSOC group between baseline and 24 months was small and not statistically significant (Δ_T1–T0_ = 1.14, *p* = 0.711). In contrast, there was a statistically significant increase in the proportion of virally suppressed participants in the intervention group (Δ_T1–T0_ = 9.99, *p* = 0.001). Overall, the intervention was associated with a statistically significant 8.85‐percentage point [95% confidence interval (CI) 0.80 to 16.90 percentage points] difference in the proportion of virally suppressed adolescents across the study arms at 24 months (*p* = 0.032). Intervention effects disaggregated by gender and age are presented (Tables S5‐S8) in Supplementary Material. Furthermore, TOT analysis showed a statistically significant 8.43‐percentage point increase in the proportion of virally suppressed adolescents across study arms at 24 months (*p* = 0.039) (Table S9), which is slightly lower but similar to the increase estimated in ITT analysis.

**Table 1:** Difference‐in‐differences analysis of change in the proportion of virally suppressed adolescents (<40 copies/mL) based on ITT sample by study arm

	BSOC arm				Intervention arm				Difference		
	T_0_	T_1_	Δ_T1–T0_	*p*‐value	T_0_	T_1_	Δ_T1–T0_	*p*‐value	Δ_T1–T0 (Intervention)_‐ Δ_T1–T0 (BSOC)_	SE	*p*‐value
Sample size	344	322	–	–	358	331	–	–	–	–	–
n virally suppressed	214	203	–	–	200	218	–	–	–	–	–
% virally suppressed	62.21	63.35	1.14	0.711	55.87	65.86	9.99	0.001	8.85	0.04	0.032

BSOC, Bolstered standard of care; SE, standard error; T_0_, Baseline; T_1_, 24 months.

### 3.3 Costs and cost‐effectiveness

Table 2 presents the total per‐participant costs for each study arm based on the TOT sample. The total per‐participant cost was US$177 for the BSOC group, and US$263 for the intervention group. Total per‐participant costs estimated based on the ITT sample (Table S3) and costs per virally suppressed adolescent by study arm (Table S4) are presented in Supplementary Material.

**Table 2:** Total per‐participant costs by study arm (all costs in Ugandan Shillings for the year 2015 unless otherwise noted)

Costs	BSOC arm	Intervention arm
Personnel (salaries)	12,549	25,101
Recruitment of participants	15,411	16,040
Bolstered standard of care	516,993	511,484
Health education sessions and mentorship	–	98,927
Microenterprise workshops	–	43,138
Child savings account	–	128,885
Initial deposit	–	20,000
Account opening	–	32,214
Matched contributions	–	76,671
Monitoring and evaluation	22,726	26,878
Stakeholder engagement and dissemination	8452	5524
Total per‐participant cost	576,132	855,977
Total per‐participant cost (in 2015 USD)	177	263

BSOC, Bolstered standard of care.

Given the mean net change of 8.85 percentage points in the proportion of virally suppressed adolescents across the study arms at 24 months, the intervention was estimated to virally suppress, on average, an additional 32 adolescents over this period. The per‐participant cost difference between the two arms was US$86 over the same period. Hence, the incremental cost of virally suppressing one additional adolescent was estimated at US$970 [95% CI, US$508 to 10,725]. Under the optimistic and pessimistic scenarios, ICERs ranged from US$408 to US$12,875 per additional virally suppressed adolescent.

## 4 Discussion

ART scale‐up for PLWHA, including ALWHIV, has been a global priority for over a decade. However, lifelong adherence to treatment has proved to be a major barrier to actualizing the full potential of ART [30]. Complexity and side‐effects of ART regimens contribute to suboptimal adherence, both intentional and non‐intentional [12], that leads to poor health outcomes [12, 16, 21, 31]. Adherence‐support interventions tailored to the social and economic contexts in which they are implemented are now considered critical to the success and sustainability of HIV programmes [13, 14, 16].

Our study contributes evidence not only on the efficacy but also on the costs and cost‐effectiveness of a savings‐led family‐based EE intervention to improve adherence outcomes among ALWHIV. Our analysis showed that the incorporation of EE interventions into BSOC would increase the proportion of virally suppressed adolescents by about 9% while increasing the cost of providing adherence support per adolescent by about 50% (US$177 versus US$263 per adolescent) over two years. Based on the total costs of providing BSOC and Suubi + Adherence intervention, the cost per virally suppressed adolescent was estimated at US$249 and US$361 for the control and intervention groups, respectively, over the intervention period (Table S4). Only two other trials have to date assessed interventions aimed at improving viral suppression among ALWHIV in low‐resource settings [14, 15], but only one trial conducted a cost analysis of the intervention [15]. This trial tested a peer‐supported differentiated service delivery intervention for adolescents in Zimbabwe, involving monthly support groups, monthly home visits, daily or weekly text message reminders, weekly phone calls, health centre contact and caregiver workshops, and estimated a substantially higher cost of US$1340 per virally suppressed adolescent per year while reporting a 42% lower prevalence of virological failure using a cutoff of 1,000 copies per mL or death at 96 weeks among participating ALWHIV [15]. In resource‐poor countries like Uganda, intervention costs are critical for the translation of research findings into real‐world settings. If we consider scale‐up to a larger target population, the actual cost of replicating Suubi + Adherence intervention would likely be lower than the costs reported here. For example some fixed costs associated with the delivery of the intervention would be spread over a larger denominator. However, matched funds and conditionality on their use would likely impose a complex administrative and financial layer. There are several other locally supported programmes promoting microfinance for youth in SSA and other low‐resource settings [32]. Matched funds and financial education are generally provided by non‐profit and government sources, and CDAs are housed at local financial institutions. In the case of Uganda, EE interventions could be incorporated within government‐ and NGO‐led youth‐focused interventions. Indeed, savings‐led interventions promoted via CDAs are in line with the Ugandan Government’s Vision 2040, which calls for investment in financial inclusion for the most vulnerable groups [33]. It is unclear if intervention costs such as these are acceptable to policymakers in low‐resource settings. Yet, despite high costs, particularly in the form of matching funds, savings‐led EE interventions equip youth with skills and resources needed for self‐sustenance, reducing their dependence on government or foreign aid in the long‐run. Nonetheless, there is a need to develop funding mechanisms to complement, improve or simply generate resources for such interventions to operate optimally at scales necessary to meet the demands of this vulnerable population [32].

Moreover, in resource‐constrained settings, the cost‐effectiveness of interventions is crucial to assess their sustainability. Overall, a few RCTs, mostly conducted in high‐resource settings, have evaluated the cost‐effectiveness of adherence interventions in general [34], and there is a dire lack of trial‐based economic evaluations of ART adherence interventions in low‐resource settings in particular. Our cost‐effectiveness analysis of the Suubi + Adherence intervention showed that it would, on average, cost US$970 to virally suppress one additional ALWHIV relative to BSOC over two years. Further research is warranted to help establish cost‐effectiveness benchmarks in low‐resource settings that can guide researchers in intervention development, and policymakers in policy formulation and analysis. Overall, our results are in line with studies from low‐resource settings showing that EE interventions have the potential to improve adherence outcomes among ALWHIV by mitigating the effects of poverty [16, 29]. Our findings are also consistent with studies from high‐income countries reporting that financial incentives to promote ART adherence in low‐income populations are not only effective, but can also be cost‐effective in suppressing VL in HIV patients [35].

As HIV treatment expanded rapidly in countries heavily burdened by HIV/AIDS, several studies highlighted the economic inefficiency associated with providing high‐cost treatment with limited effectiveness due to low levels of ART adherence. These studies have argued that investing a significant proportion of programme resources in monitoring and improving ART adherence is justified [36, 37]. Early detection of and response to suboptimal ART adherence not only leads to improved immunological recovery (higher CD4+ cell counts) and better health outcomes in patients but also preserves the effectiveness of less costly first‐line ART regimens, reducing the need for more expensive second‐line treatments in settings where healthcare resources and therapeutic options are limited [38]. In fact, a recent study on the long‐term effects of Suubi + Adherence intervention showed a significantly higher incidence of undetectable VL among participants in the intervention group compared to those in the non‐intervention group, pointing to the potential role of EE interventions in assisting with HIV care and retention [29]. Viral suppression has been associated with long‐term improvements in labour productivity and economic stability, including via improved educational outcomes in childhood. Given the disproportionately poor HIV outcomes among ALWHIV, and considering the economic consequences of suboptimal adherence, from a policy standpoint, donors and decision makers may be justified in investing in resource‐intensive adherence‐support interventions for ALWHIV. Studies in low‐income countries have shown that there are great cost‐inefficiencies in HIV service provision, and streamlining services through a tailored approach, for example based on patients’ immunological status and psychosocial characteristics, is shown to free up resources [39] that could potentially offset the costs of community‐based ART adherence interventions for the most vulnerable populations, such as ALWHIV, as recommended by the WHO.

The study findings should be considered in light of several limitations. First, we used a very conservative cutoff value of HIV RNA 40 copies/mL for viral suppression. Further research is needed to understand the effect of using other less conservative but clinically relevant cutoff points on cost‐effectiveness results. Second, the intervention was tailored to a very specific population: ALWHIV in rural Uganda. Therefore, our findings may not be generalizable to other age groups or income settings. VL is considered the most reliable indicator of treatment adherence at the population level [38]; however, it is not a clinical disease outcome, such as mortality. Nonetheless, viral suppression as a direct intervention effect is correlated with improved quality of life, longer survival and reduced onward transmission, and its use in an economic evaluation is, therefore, justified [35]. Third, the cost‐effectiveness analysis was conducted from a healthcare provider perspective. Although participants received incentives for participating in the trial, out‐of‐pocket expenses for transportation to health centres and time costs of seeking HIV care were not systematically measured and explicitly considered in the analysis. On the other hand, the potential benefits of the intervention in terms of improved health outcomes and increased productivity were also not included. Future economic evaluations could use a broader perspective to consider these potential costs and benefits accruing to participants and the community at large. Lastly, a review of high‐quality adherence trials reported that effects on adherence indicators or surrogate clinical outcomes were usually assessed at six to twelve months, providing limited understanding of their sustainability [11]; our study partially overcomes this limitation by providing data from a follow‐up period of 24 months. However, future cost‐effectiveness analyses with an extended time horizon are warranted for the consideration of clinical disease outcomes.

A strength is that the study design addresses and controls for multiple biases found in past economic evaluations of interventions aimed at improving ART adherence in HIV patients. First, intervention efficacy was assessed using data collected in the context of an RCT, thus minimizing the risk of selection and allocation biases. In addition, an objective and reliable indicator of adherence was used to assess efficacy, rather than relying on self‐reported measures. Loss‐to‐follow‐up was low and did not differ significantly between the two study arms, and hence was unlikely to have influenced the study conclusions. Another strength is that cost data were collected prospectively alongside the trial, minimizing errors and recall biases. Costing tools were developed *a priori* and programme staff received training on their completion. Nevertheless, further research is warranted to replicate and build on these findings in similar intervention settings.

## 5 Conclusions

Our findings add to the growing evidence base on EE interventions in improving ART adherence outcomes among ALWHIV and provide further justification for their integration into adherence‐support strategies as part of routine HIV care in low‐resource settings. Our study also addresses an identified evidence gap on the costs and cost‐effectiveness of adherence interventions in the context of RCTs [34] and contributes to the establishment of cost‐effectiveness benchmarks for behavioural trials in such settings. Further research and policy discussion on combination interventions at the nexus of economic security and HIV treatment and care is needed to inform the development of feasible and scalable HIV policies and programmes.

## Competing interests

The authors have declared no conflict of interest.

## Authors’ contributions

YT conceived and designed the cost‐effectiveness study with FMS, and wrote the manuscript. AC and SS analysed the cost and efficacy data with input from YT and TBN. CD, FV, GN, AM and BM contributed to the collection of trial data used in the study. OSB and PN contributed to the interpretation of results. FMS conceptualized and designed the trial with input from CAM and MMM, and was responsible for its supervision and funding acquisition. All authors contributed to the revisions of the manuscript and approved the final version.

## Supporting information


**Figure S1**. CONSORT Flow Diagram: Suubi + Adherence Study.
**Table S1**. Characteristics of 702 adolescents at baseline by study arm
**Table S2**. Cost calculation methods
**Table S3**. Total per‐child costs by study arm using ITT sample
**Table S4**. Costs per virally suppressed adolescents by study arm
**Table S5**. Difference‐in‐differences analysis of change in the proportion of virally suppressed adolescents (<40 copies/mL) based on ITT sample by study arm, male participants
**Table S6**. Difference‐in‐differences analysis of change in the proportion of virally suppressed adolescents (<40 copies/mL) based on ITT sample by study arm, female participants
**Table S7**. Difference‐in‐differences analysis of change in the proportion of virally suppressed adolescents (<40 copies/mL) based on ITT sample by study arm, age 10 to 12 years
**Table S8**. Difference‐in‐differences analysis of change in the proportion of virally suppressed adolescents (<40 copies/mL) based on ITT sample by study arm, age 13 to 16 years
**Table S2**. Difference‐in‐differences analysis of change in the proportion of virally suppressed adolescents (<40 copies/mL) based on TOT sample by study arm
