# Supplementary material for: The efficacy and cost‐effectiveness of a family‐based economic empowerment intervention (Suubi + Adherence) on suppression of HIV viral loads among adolescents living with HIV: results from a Cluster Randomized Controlled Trial in southern Uganda
Source: J Int AIDS Soc. 2021 Jun 27;24(6):e25752. doi: 10.1002/jia2.25752 (PMC8236226; doi:10.1002/jia2.25752)
Supplement: Supplementary file 1 — Figure S1. CONSORT Flow Diagram: Suubi + Adherence Study. Table S1. Characteristics of 702 adolescents at baseline by study arm Table S2. Cost calculation methods Table S3. Total per‐child costs by study arm using ITT sample Table S4. Costs per virally suppressed adolescents by study arm Table S5. Difference‐in‐differences analysis of change in the proportion of virally suppressed adolescents (<40 copies/mL) based on ITT sample by study arm, male participants Table S6. Difference‐in‐differences analysis of change in the proportion of virally suppressed adolescents (<40 copies/mL) based on ITT sample by study arm, female participants Table S7. Difference‐in‐differences analysis of change in the proportion of virally suppressed adolescents (<40 copies/mL) based on ITT sample by study arm, age 10 to 12 years Table S8. Difference‐in‐differences analysis of change in the proportion of virally suppressed adolescents (<40 copies/mL) based on ITT sample by study arm, age 13 to 16 years Table S2. Difference‐in‐differences analysis of change in the proportion of virally suppressed adolescents (<40 copies/mL) based on TOT sample by study arm [file JIA2-24-e25752-s001.docx]

**Supplementary material**


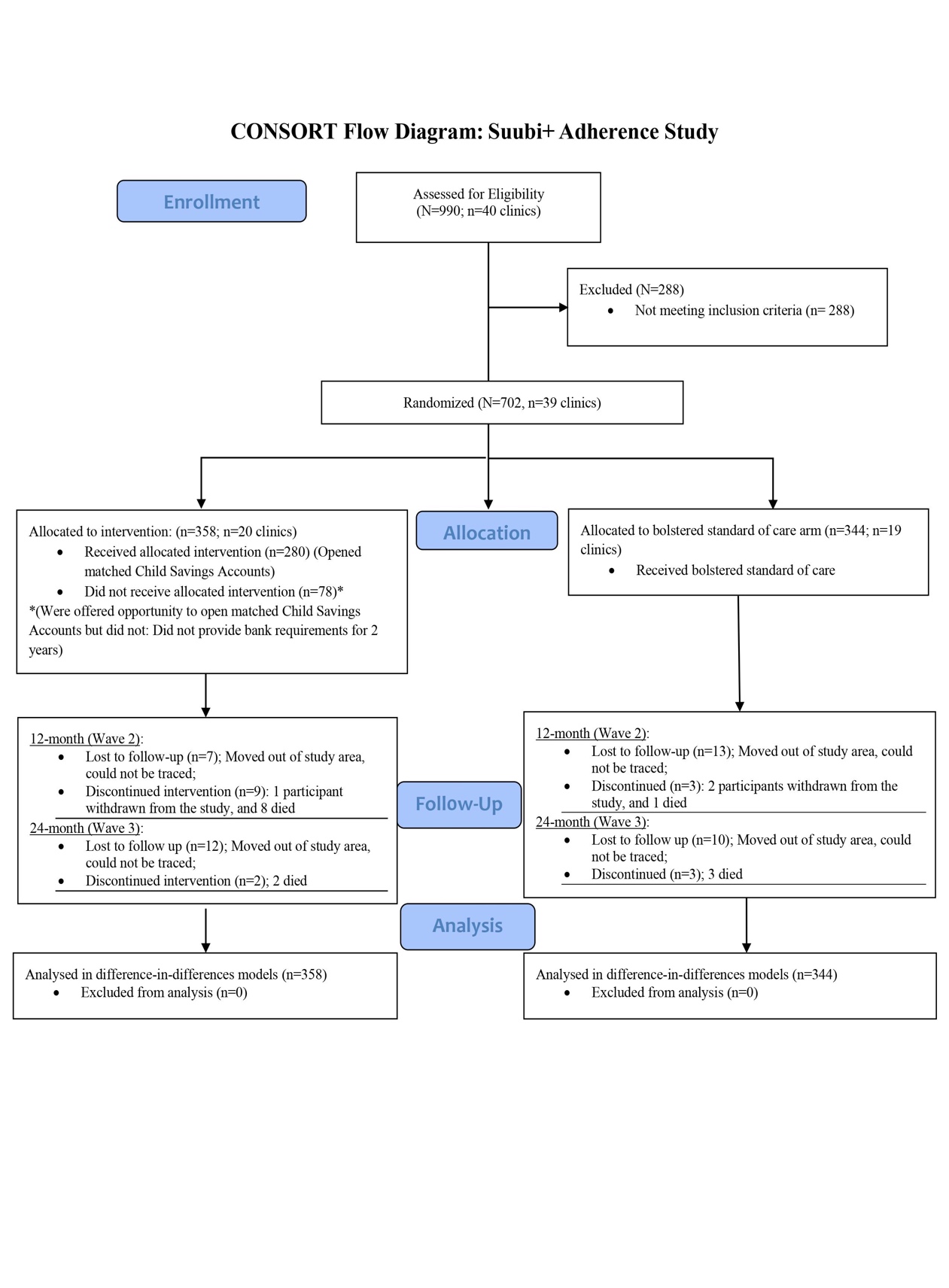


Figure S1. CONSORT Flow Diagram: Suubi+Adherence Study

*Notes*: The study uses an intent‐to‐treat design. Therefore, adolescents who did not respond to the 12‐month interview were tracked and followed up at 24-months. Thus, the lost‐to‐follow‐up numbers at 12-months and 24-months post‐intervention initiation are relative to the baseline number.

Table S1. Characteristics of 702 adolescents at baseline by study arm

|  | **BSOC arm**  (n = 344) | **Intervention arm**  (n = 358) | **Total**  (n = 702) |
| --- | --- | --- | --- |
| **Socio-demographic characteristics** |  |  |  |
| Age | 12.38 (1.97) | 12.46 (1.98) | 12.42 (1.98) |
| Gender |  |  |  |
| Male | 151 (43.90%) | 155 (43.30%) | 306 (43.59%) |
| Female | 193 (56.10%) | 203 (56.70%) | 396 (56.41%) |
| Household size | 5.78 (2.46) | 5.70 (2.65) | 5.74 (2.56) |
| Number of children in household | 2.41 (1.88) | 2.29 (1.97) | 2.35 (1.92) |
| Orphan status |  |  |  |
| Both alive | 116 (33.72%) | 133 (37.15%) | 249 (35.47%) |
| Single orphan | 133 (38.66%) | 138 (38.55%) | 271 (38.60%) |
| Double orphan | 95 (27.62%) | 87 (24.30%) | 182 (25.93%) |
| Years since living in the household | 8.87 (4.25) | 9.19 (4.13) | 9.03 (4.19) |
| Primary caregiver |  |  |  |
| Parent(s) | 151 (43.90%) | 179 (50.14%) | 330 (47.08%) |
| Grandparent(s) | 102 (29.65%) | 104 (29.13%) | 206 (29.39%) |
| Others | 91 (26.45%) | 74 (20.73%) | 165 (23.54%) |
| Current schooling |  |  |  |
| In school | 301 (87.5%) | 312 (87.15%) | 613 (87.32%) |
| Not in school | 43 (12.5%) | 46 (12.85%) | 89 (12.68%) |
| **HIV-related characteristics** |  |  |  |
| Viral load suppression |  |  |  |
| Suppressed (< 40 copies/ml) | 214 (62.21%) | 200 (55.87%) | 414 (58.97%) |
| Unsuppressed (≥ 40 copies/ml) | 130 (37.79%) | 158 (44.13%) | 288 (41.03%) |
| Number of pills in daily regimen | 1.95 (0.69) | 2.06 (0.69) | 2.01 (0.68) |
| Years since knew HIV status | 4.23 (2.86) | 4.13 (3.04) | 4.18 (2.95) |
| **Sample size** |  |  |  |
| Baseline | 344 | 358 | 702 |
| 24-months follow-up | 322 | 331 | 653 |

Note: n (%) are provided for categorical variables and mean (standard deviation) are provided for continuous variables.

Table S2. Cost calculation methods

| **Costs** | **Cost items** | **Per-person cost calculation methods** |
| --- | --- | --- |
| Program staff | Based on administrative records, time devoted to program activities by staff | Extract the number of hours each staff member devoted to program activities each year (as opposed to research), multiply the total hours by average hourly salary rate for staff, and calculate the total cost across all staff. Apportion the total cost incurred each year based on level of effort dedicated by staff to program activities in each arm (33% BSOC arm, 67% intervention arm) and divide by the number of participants in each arm. |
| Recruitment of participants | Communication costs to health clinics (airtime), fuel to travel to health clinics, time spent for recruitment activities by community partners | Divide the total cost incurred by the number of participants recruited to each arm. |
| Bolstered standard of care | Training of lay counselors and expert clients, translation and printing of educational materials, fuel to travel to health clinics, meals and refreshments, transport refund for participants, facilitation incentives for lay counselors and expert clients, communications costs, donated health clinic space, and donated time by lay counselors and expert clients | Divide the total cost incurred by the number of participants in each arm. |
| Health education sessions and mentorship | Translation and printing of education materials, fuel to travel to health clinics, transport refund for participants, snacks and refreshments, facilitation incentives for contact lay counselors and clinic directors, communications costs, donated health clinic space, and donated time by lay counselors | Divide the total cost incurred each year by the number of participants in the intervention arm. |
| Microenterprise workshops | Fuel to travel to health clinics, car hire, transportation refund for participants, snacks and refreshments, stationery, printing of handouts, participation incentives for extension workers, facilitation incentives for contact lay counselors, lunch for program staff, donated space at health clinics, and donated time by lay counselors | Divide the total cost incurred each year by the number of participants in the intervention arm. |
| Child development account – Bank account opening | Fuel to travel to health clinics, transportation refund for participants, printing of account opening forms, facilitation incentives for lay counselors, donated time by bank officials, initial deposit to bank accounts (5,600,000 UGX), lunch for program staff | Divide the total cost incurred each year by the number of participants in the intervention arm. |
| Child development account – Matched contributions | Total amount of funds matched by the study for the entire intervention period |  |
| Program monitoring and evaluation | Training of program staff, fuel for travel to health clinics, car hire, meals and refreshments, travel refund and participation incentives for contact lay counselors, expert clients and clinic directors | Apportion the total cost incurred each year based on level of effort dedicated by staff to program activities in each arm and divide by the number of participants in each study arm. |
| Stakeholder engagement and dissemination | Donated time by community partners to participate in presentations to introduce the project, provide updates and disseminate the findings to the community and donated space | Estimate and monetize time spent by community partners in program-related workshops and meetings each year, apportion the total cost based on level of effort devoted by staff to program activities in each arm, and divide by the number of participants in each study arm. |

Table S3. Total per-child costs by study arm using ITT sample

(All costs are in 2015 Ugandan Shillings unless otherwise indicated)

| **Costs** | **BSOC arm** | **Intervention arm** |
| --- | --- | --- |
| Personnel (salaries) | 12,549 | 25,101 |
| Recruitment of participants | 15,411 | 16,040 |
| Bolstered standard of care | 384,291 | 405,925 |
| Health education sessions and mentorship | - | 55,543 |
| Microenterprise workshops | - | 33,081 |
| Child savings account | - | - |
| *Initial deposit* | - | 20,000 |
| *Account opening* | - | 20,838 |
| *Matched contributions* | - | 59,966 |
| Monitoring and evaluation | 15,181 | 19,657 |
| Stakeholder engagement and dissemination | 8,452 | 5,524 |
| Total costs | 435,885 | 661,674 |
| **Total costs (in 2015 USD)** | **134** | **204** |

Table S4. Costs per virally suppressed adolescents by study arm

(All costs in Ugandan Shillings for the year 2015 unless otherwise noted)

|  | **BSOC arm** | **Intervention arm** |
| --- | --- | --- |
| Total program cost over 24 months | 164,289,859 | 253,312,983 |
| Virally suppressed adolescents at 24-months, n | 203 | 218 |
| Cost per virally suppressed adolescent | 809,309.65 | 1,171,160.47 |
| **Cost per virally suppressed adolescent (in 2015 USD)** | **249** | **361** |

Table S5. Difference-in-differences analysis of change in the proportion of virally suppressed adolescents (<40 copies/ml) based on ITT sample by study arm, male participants

|  | BSOC arm | | | | Intervention arm | | | | Difference | | |
| --- | --- | --- | --- | --- | --- | --- | --- | --- | --- | --- | --- |
|  | T_0_ | T_1_ | Δ_T1 – T0_ | p-value | T_0_ | T_1_ | Δ_T1 – T0_ | p-value | Δ_T1 – T0_ _(Intervention)_- Δ_T1 – T0_ _(BSOC)_ | S.E. | p-value |
| Sample size | 151 | 147 | - | - | 155 | 145 | - | - | - | - | - |
| n virally suppressed | 99 | 95 | - | - | 78 | 93 | - | - | - | - | - |
| % virally suppressed | 65.56 | 64.63 | -0.93 | 0.757 | 50.32 | 64.14 | 13.82 | 0.0002 | 14.75 | 0.04 | 0.001 |

BSOC = Bolstered standard of care; S.E. = Standard Error; T_0_ = Baseline; T_1_ = 24 months

Table S6. Difference-in-differences analysis of change in the proportion of virally suppressed adolescents (<40 copies/ml) based on ITT sample by study arm, female participants

|  | BSOC arm | | | | Intervention arm | | | | Difference | | |
| --- | --- | --- | --- | --- | --- | --- | --- | --- | --- | --- | --- |
|  | T_0_ | T_1_ | Δ_T1 – T0_ | p-value | T_0_ | T_1_ | Δ_T1 – T0_ | p-value | Δ_T1 – T0_ _(Intervention)_- Δ_T1 – T0_ _(BSOC)_ | S.E. | p-value |
| Sample size | 193 | 175 | - | - | 203 | 186 | - | - | - | - | - |
| n virally suppressed | 115 | 109 | - | - | 122 | 125 | - | - | - | - | - |
| % virally suppressed | 59.59 | 62.29 | 2.70 | 0.613 | 60.10 | 67.20 | 7.10 | 0.135 | 4.41 | 0.07 | 0.527 |

BSOC = Bolstered standard of care; S.E. = Standard Error; T_0_ = Baseline; T_1_ = 24 months

Table S7. Difference-in-differences analysis of change in the proportion of virally suppressed adolescents (<40 copies/ml) based on ITT sample by study arm, age 10-12 years

|  | BSOC arm | | | | Intervention arm | | | | Difference | | |
| --- | --- | --- | --- | --- | --- | --- | --- | --- | --- | --- | --- |
|  | T_0_ | T_1_ | Δ_T1 – T0_ | p-value | T_0_ | T_1_ | Δ_T1 – T0_ | p-value | Δ_T1 – T0_ _(Intervention)_- Δ_T1 – T0_ _(BSOC)_ | S.E. | p-value |
| Sample size | 184 | 177 | - | - | 106 | 172 | - | - | - | - | - |
| n virally suppressed | 113 | 110 | - | - | 181 | 115 | - | - | - | - | - |
| % virally suppressed | 61.41 | 62.15 | 0.74 | 0.824 | 58.56 | 66.86 | 8.30 | 0.021 | 7.56 | 0.05 | 0.106 |

BSOC = Bolstered standard of care; S.E. = Standard Error; T_0_ = Baseline; T_1_ = 24 months

Table S8. Difference-in-differences analysis of change in the proportion of virally suppressed adolescents (<40 copies/ml) based on ITT sample by study arm, age 13-16 years

|  | BSOC arm | | | | Intervention arm | | | | Difference | | |
| --- | --- | --- | --- | --- | --- | --- | --- | --- | --- | --- | --- |
|  | T_0_ | T_1_ | Δ_T1 – T0_ | p-value | T_0_ | T_1_ | Δ_T1 – T0_ | p-value | Δ_T1 – T0_ _(Intervention)_- Δ_T1 – T0_ _(BSOC)_ | S.E. | p-value |
| Sample size | 160 | 145 | - | - | 177 | 159 | - | - | - | - | - |
| n virally suppressed | 101 | 94 | - | - | 94 | 103 | - | - | - | - | - |
| % virally suppressed | 63.13 | 64.83 | 1.70 | 0.614 | 53.11 | 64.78 | 11.67 | 0.0004 | 9.97 | 0.04 | 0.025 |

BSOC = Bolstered standard of care; S.E. = Standard Error; T_0_ = Baseline; T_1_ = 24 months

Table S9. Difference-in-differences analysis of change in the proportion of virally suppressed adolescents (<40 copies/ml) based on TOT sample by study arm

|  | BSOC arm | | | | Intervention arm | | | | Difference |  |  |
| --- | --- | --- | --- | --- | --- | --- | --- | --- | --- | --- | --- |
|  | T_0_ | T_1_ | Δ_T1 – T0_ | p-value | T_0_ | T_1_ | Δ_T1 – T0_ | p-value | Δ_T1 – T0_ _(Intervention)_- Δ_T1 – T0_ _(BSOC)_ | S.E. | p-value |
| Sample size | 344 | 322 | - | - | 280 | 270 | - | - | - | - | - |
| n virally suppressed | 214 | 204 | - | - | 164 | 184 | - | - | - | - | - |
| % virally suppressed | 62.21 | 63.35 | 1.14 | 0.711 | 58.57 | 68.15 | 9.58 | 0.001 | 8.43 | 0.04 | 0.039 |

BSOC = Bolstered standard of care; S.E. = Standard Error; T_0_ = Baseline; T_1_ = 24 months
